# Exponential Phosphorus Application Enhances Growth, Photosynthesis and Nutrient Accumulation of *Homalium ceylanicum* Seedlings

**DOI:** 10.3390/plants15152316

**Published:** 2026-07-28

**Authors:** Ning Ma, Zhiqiang Fu, Daping Xu, Zhou Hong, Zhiyi Cui, Baohua Wang, Yu Su, Xiaojin Liu

**Affiliations:** 1 Research Institute of Tropical Forestry, Chinese Academy of Forestry, Guangzhou 510520, China; mning1007@163.com (N.M.);; 2 College of Landscape Architecture, Nanjing Forestry University, Nanjing 210037, China; 3 Yangjiang State-Owned Forest Farm, Yangjiang 529500, China; 4 Guangzhou Institute of Forestry and Landscape Architecture, Guangzhou 510080, China

**Keywords:** *Homalium ceylanicum*, nutrient loading, phosphorus, photosynthetic characteristics, vector analysis

## Abstract

Phosphorus (P) deficiency is a major nutritional constraint limiting the regeneration and large-scale cultivation of *Homalium ceylanicum*, a valuable tropical timber species in South and Southeast Asia. To identify an optimal P supply strategy for producing high-quality planting stock, we assessed the growth performance, photosynthetic characteristics, and nutrient status of one-year-old *H. ceylanicum* seedlings in response to exponential P fertilization at six levels: 0 (control), 25, 50, 100, 200 and 300 mg P per seedling. Seedlings supplied with 50 mg P per seedling performed best, with height, leaf biomass, and leaf area increasing by 57.5%, 152.0%, and 164.7%, respectively, relative to the unfertilized control. These enhancements were paralleled by significant improvements in net photosynthetic rate, chlorophyll content, photosystem II (PSII) photochemical efficiency, and gas exchange, indicating that enhanced photosynthetic carbon assimilation contributed to growth promotion. At the whole-plant level, this coordinated improvement in growth and photosynthesis was accompanied by a 171.04% increase in nitrogen (N) accumulation without inducing excessive P accumulation. In contrast, P supply above 100 mg per seedling reduced seedling height and root collar diameter, suppressed whole-plant biomass accumulation, and disrupted nutrient balance through excessive P accumulation and antagonistic effects on N and K (potassium). Vector analysis further confirmed 50 mg P per seedling as the optimal nutrient-loading dose, achieving effective P loading without inducing N or K excess or nutrient imbalance. In conclusion, exponential P fertilization at 50 mg P per seedling is a targeted nutritional strategy for producing high-quality planting stock, thereby supporting the plantation forestry of *H. ceylanicum*.

## 1. Introduction

Phosphorus (P) is an essential macronutrient that plays a vital role in plant growth and development by participating in numerous fundamental physiological processes, including photosynthesis, energy metabolism, cell division, and biosynthesis [1]. Adequate P supply enhances leaf photosynthetic performance, increases chlorophyll content, and significantly improves the maximum photochemical quantum yield of photosystem II in various tree species [2,3,4]. Concurrently, it promotes seedling growth in terms of height, root collar diameter (RCD), leaf area, and biomass accumulation while modulating the uptake and allocation of other key nutrients such as nitrogen (N) and potassium (K) [5,6,7]. However, P requirements are highly species-specific, and both insufficient and excessive application can be detrimental [8]. For instance, seedlings of *Eucalyptus dunnii* and *Corymbia citriodora* exhibited optimal growth under hydroponic culture at P concentrations of 0.1–1.0 mmol L^−1^, whereas both lower (0–0.01 mmol L^−1^) and excessive (2.0 mmol L^−1^) P supplies resulted in stunted growth [9]. Similarly, a study on two-year-old *Bertholletia excelsa* seedlings demonstrated that key physiological parameters, including net photosynthetic rate, stomatal conductance, and transpiration rate, peaked at a soil P concentration of 200 mg kg^−1^, with other P levels generally showing less favorable physiological responses [10]. Furthermore, the efficacy of P fertilization depends not only on the application rate but also on the application method [11]. Conventional practice often relies on fertilization with a constant amount; however, this approach may not adequately align with the exponential growth dynamics of seedlings, leading to an initial oversupply followed by a subsequent deficiency of P [12]. Therefore, adopting a phased application strategy that matches the species-specific P demands with the exponential growth pattern of seedlings is essential for optimizing physiological processes and producing high-quality seedlings.

Exponential fertilization, based on the principle of steady-state nutrition, supplies nutrients at progressively increasing rates to better synchronize nutrient availability with seedling growth and promote nutrient loading [13]. This strategy has been successfully applied to a range of tree species [14,15,16,17]. However, effective nutrient loading requires increasing internal nutrient reserves while sustaining biomass production [18]. Vector analysis provides a valuable diagnostic approach for this purpose, as it can simultaneously integrate biomass, nutrient concentration, and nutrient content to distinguish among deficiency alleviation, sufficiency, luxury consumption, and toxicity-like responses. Chen et al. found that 100–400 mg N seedling^−1^ induced N responses ranging from sufficiency to luxury consumption in *Betula alnoides* seedlings, with 400 mg N seedling^−1^ recommended for nutrient loading before outplanting [19]. Feng et al. showed that, under exponential fertilization in *Pinus armandii* seedlings, 300 mg N per seedling induced luxury consumption, whereas 400 mg N per seedling resulted in toxicity-like responses [12]. Similarly, Uscola et al. defined 125 mg N per seedling as the sufficiency level and 200 mg N per seedling as the luxury-consumption level in *Quercus ilex* seedlings, with no toxicity observed at the highest fertilization rate [20]. In summary, most nutrient-loading studies have focused on N supply, whereas the diagnosis of P-loading responses remains relatively limited, especially in tropical broad-leaved tree seedlings. This gap is critical because P may shift from a limiting nutrient to an excessive input within a comparatively narrow application range [21,22]. In particular, the total P input required to achieve effective P loading without antagonistic changes in N and K accumulation remains unclear.

*Homalium ceylanicum,* belonging to the family Salicaceae, is a valuable tropical timber species naturally distributed across South and Southeast Asia [23]. It is valued for its superior wood quality, including fine texture, high density, high strength, and resistance to deformation, making it suitable for construction, furniture manufacturing, and other high-value timber applications. However, large-scale development of *H. ceylanicum* plantations is constrained by low-quality planting stock, which results in a low planting survival rate, and improved nursery cultivation techniques are urgently needed for this species [24,25]. Moreover, the native soils where *H. ceylanicum* grows are generally P-deficient [26,27]. Therefore, addressing this P limitation through targeted fertilization strategies and elucidating the underlying physiological responses are essential to produce high-quality planting stock. Accordingly, this study aimed to investigate the effects of different exponential P fertilization rates on growth performance, photosynthetic characteristics, and nutrient accumulation in one-year-old *H. ceylanicum* seedlings. The findings are expected to provide insights for optimizing seedling cultivation protocols and supporting the plantation forestry of this tree species.

## 2. Results

### 2.1. Growth Performance

**Figure 1:**
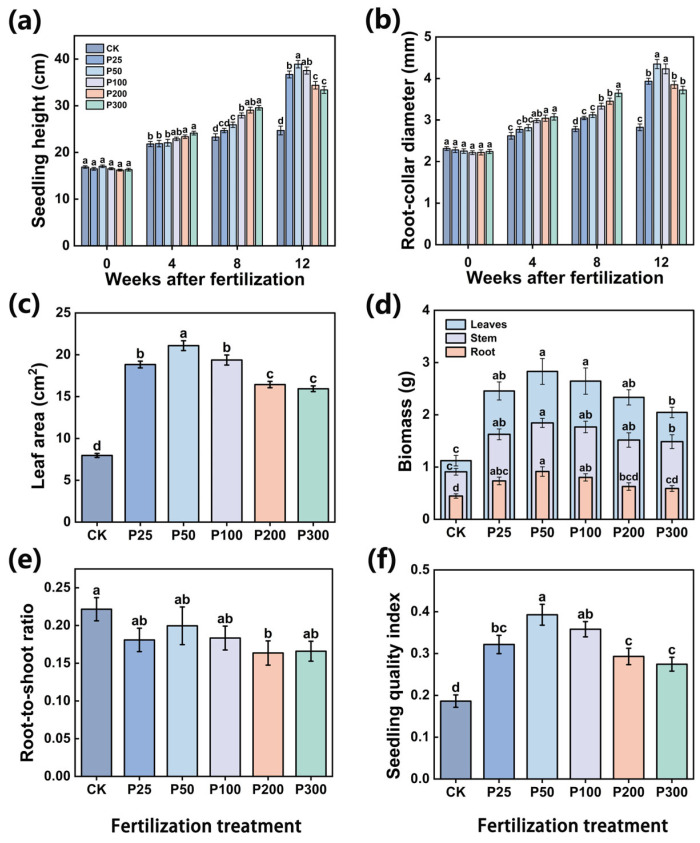
Effects of different P application rates on seedling height (**a**), root collar diameter (**b**), leaf area (**c**), leaf, stem, and root biomass (**d**), root-to-shoot ratio (**e**), and seedling quality index (**f**) of *Homalium ceylanicum* seedlings. Data are presented as mean ± SE (error bars). Different lowercase letters indicate significant differences among treatments within the same measurement time or panel according to Tukey’s test at the 0.05 level.

Seedling height and root collar diameter (RCD) increased progressively throughout the experimental period in all treatments (Figure 1a,b). Significant differences among treatments emerged after four weeks of fertilization (*p* < 0.05) and became more pronounced at 8 and 12 weeks. At the final measurement, the greatest seedling height was recorded in the P50 treatment (38.90 ± 0.80 cm), which exceeded that of the CK (24.70 ± 0.95 cm) by 57.5%; similarly, RCD peaked under P50 (4.35 ± 0.11 mm) and was lowest in the CK (2.83 ± 0.08 mm). Leaf area, biomass accumulation, and the quality index (QI) followed a similar pattern, reaching their maxima under P50 and declining at higher phosphorus levels (Figure 1c,d,f). The maximum leaf area in the P50 treatment was 164.7% greater than that in the CK. Compared with P50, leaf, stem, and root biomass in the P300 treatment decreased by 60.32%, 50.68%, and 51.26%, respectively. The root-to-shoot (R/S) ratio was highest in the CK (0.22 ± 0.02; Figure 1e), whereas QI under P50 was 110.8% greater than that in the CK.

### 2.2. Photosynthetic Characteristics

#### 2.2.1. Gas Exchange Parameters

**Figure 2:**
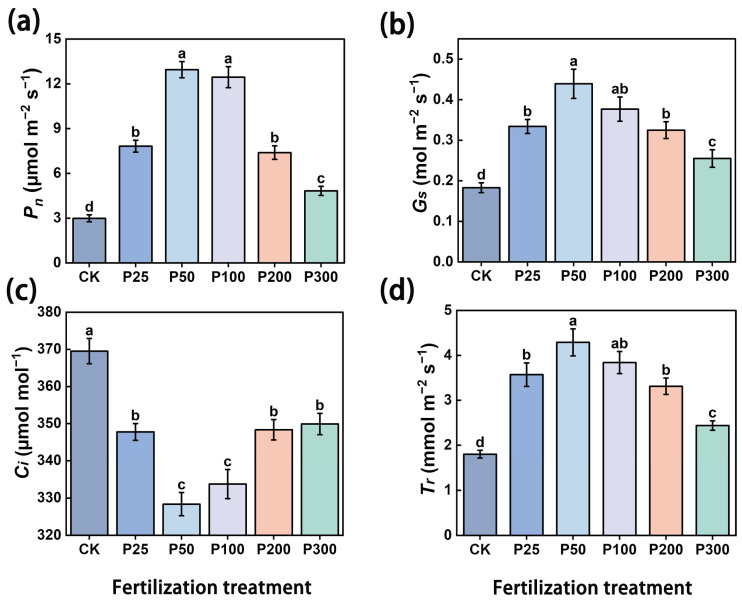
Effects of different P application rates on leaf gas exchange parameters of *Homalium ceylanicum* seedlings, including the net photosynthetic rate (**a**), stomatal conductance (**b**), intercellular CO_2_ concentration (**c**), and transpiration rate (**d**). Different lowercase letters indicate significant differences among treatments according to Tukey’s test at the 0.05 level.

Phosphorus (P) supplementation significantly influenced the net photosynthetic rate (P_n_), stomatal conductance (G_s_), intercellular CO_2_ concentration (C_i_), and transpiration rate (T_r_) (Figure 2). As the P application rate increased, P_n_, G_s_, and T_r_ generally exhibited an initial increase followed by a decline, with all three parameters reaching their maxima under the P50 treatment. Specifically, these values were 332.7%, 139.9%, and 138.3% higher than those in the CK, respectively. Higher P application levels (P200 and P300) did not further enhance these parameters but instead resulted in a reduction. In contrast, C_i_ showed the opposite trend, demonstrating significant suppression across all treatments (Figure 2c).

#### 2.2.2. Photosynthetic Pigment Content

**Figure 3:**
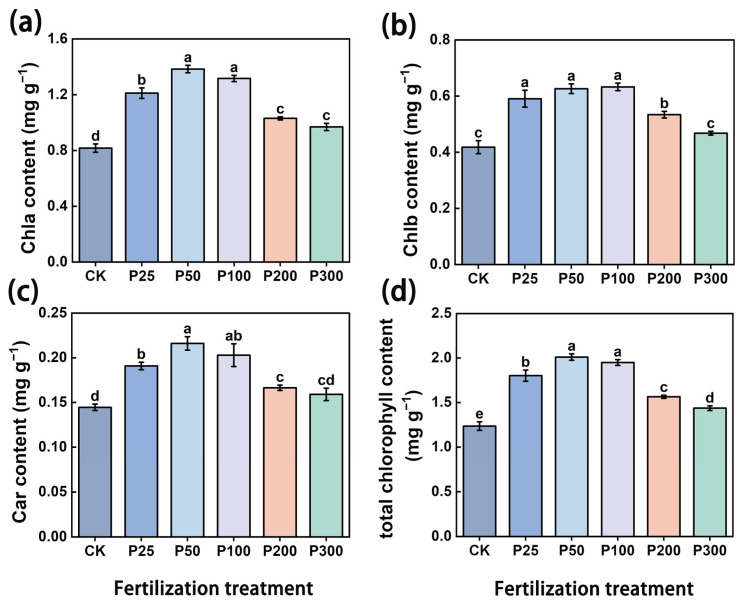
Effects of different P application rates on photosynthetic pigment contents of *Homalium ceylanicum* seedlings, showing chlorophyll a (**a**), chlorophyll b (**b**), carotenoids (**c**), and total chlorophyll (**d**). Different lowercase letters indicate significant differences among treatments according to Tukey’s test at the 0.05 level.

The contents of all photosynthetic pigments were significantly affected by P application (Figure 3). Specifically, chlorophyll a (Chl a) and carotenoid (Car) contents reached their maxima under the P50 treatment, with values of 1.38 ± 0.03 mg g^−1^ (Figure 3a) and 0.22 ± 0.01 mg g^−1^ (Figure 3c), respectively. In contrast, chlorophyll b (Chl b) content peaked under the P100 treatment (0.63 ± 0.01 mg g^−1^; Figure 3b). Total chlorophyll content (Figure 3d) was highest in the P50 treatment (2.01 ± 0.04 mg g^−1^) and lowest in the control (1.24 ± 0.05 mg g^−1^).

#### 2.2.3. Chlorophyll Fluorescence Parameters

Chlorophyll fluorescence parameters were significantly affected by P application (Table 1). The highest F_v_/F_m_ value was observed in the P50 treatment, which exceeded the values in the P300 treatment and the CK by 18.8% and 61.7%, respectively. Similar trends were observed for the effective quantum yield of PSII [Y(II)] and the electron transport rate (ETR), both of which were highest under P50 and lowest in the CK and differed markedly from the other four treatments. Photochemical quenching (q_P_) was highest under P50 and significantly exceeded the values under the CK and P25 but did not differ significantly from those under P100, P200, or P300. In contrast, non-photochemical quenching (NPQ) exhibited a different pattern. Its maximum was observed in the P50 treatment (1.72 ± 0.01), followed by the CK (1.68 ± 0.03); both values significantly exceeded that in the P200 treatment (1.50 ± 0.06).

### 2.3. Nutrient Status

#### 2.3.1. Nutrient Status in Different Organs

Nitrogen (N) concentrations in the roots, stems, and leaves of *Homalium ceylanicum* seedlings were significantly influenced by P application (Table 2). Notably, the P300 treatment markedly increased N concentrations in the roots, stems, and leaves by 38.4%, 46.2%, and 21.0%, respectively, compared with the CK. Similarly, P concentrations generally showed significant increases in response to P addition across all tissues. The maximum P concentrations were also observed in the P300 treatment, which were 195.1%, 437.7%, and 175.5% higher than those in the CK for roots, stems, and leaves, respectively. Potassium (K) concentrations in the roots, stems, and leaves were also significantly enhanced by P application. However, no significant differences were observed across the P25 to P300 treatments in stems and leaves, and the highest K concentrations in roots, stems, and leaves were all detected in the P200 treatment.

#### 2.3.2. Whole-Plant Nutritional Status

**Figure 4:**
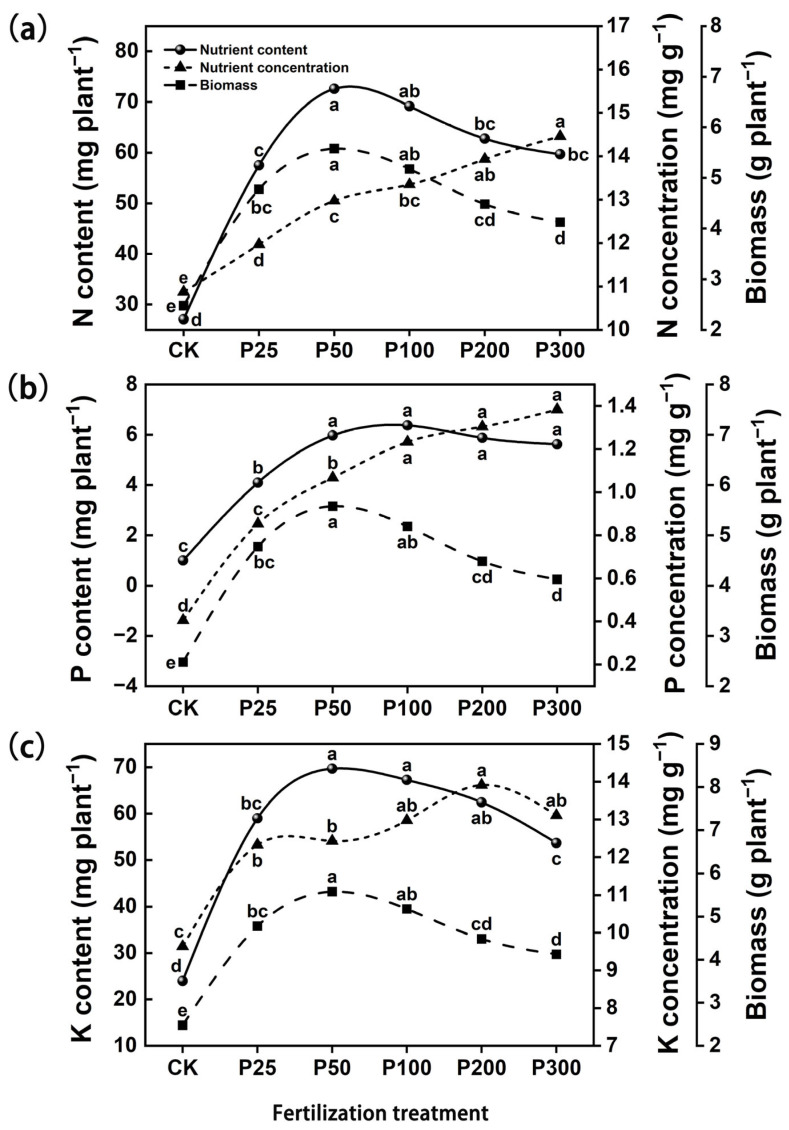
Whole-plant biomass and whole-plant N (**a**), P (**b**), and K (**c**) contents and concentrations of *Homalium ceylanicum* seedlings under different P application rates. Different lowercase letters associated with the same variable indicate significant differences among treatments according to Tukey’s test at the 0.05 level.

The response of whole-plant nutrient concentrations to increasing P supply differed from that of nutrient contents and biomass accumulation (Figure 4). Biomass accumulation and total N and K contents showed similar unimodal patterns, all peaking at P50 and declining thereafter. In contrast, total P content reached its maximum later, at P100. Whole-plant N and P concentrations continued to increase with increasing P supply and reached their highest values at P300, whereas K concentration peaked at P200 before declining at P300.

#### 2.3.3. Nutritional Diagnosis by Vector Analysis

**Figure 5:**
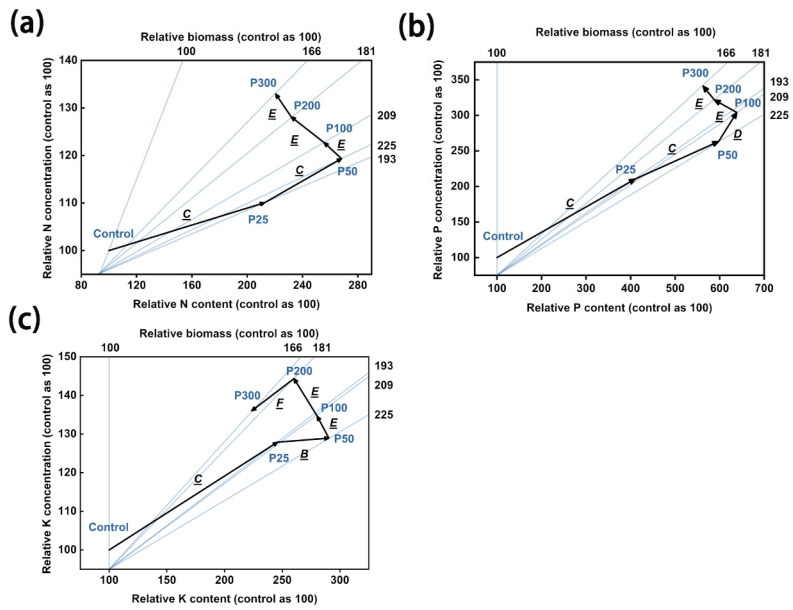
Vector analysis of relative whole-plant biomass, nutrient content, and nutrient concentration for N (**a**), P (**b**), and K (**c**) in *Homalium ceylanicum* seedlings. Values were normalized to the CK. Interpretations of vector shifts are adapted from Salifu et al. [18]. Shift B, nutrient sufficiency; Shift C, nutrient deficiency; Shift D, luxury consumption; Shift E, excess with toxic accumulation; Shift F, excess with antagonism.

The shifts in the nutritional status of N, P and K, as interpreted through vector analysis, are presented in Figure 5. From the control to the P50 treatment, biomass, N concentration, and N content increased markedly, indicating a progressive alleviation of N limitation (Shift C). Beyond P50, however, further P application resulted in elevated N concentration alongside reductions in both biomass and N content, suggesting a transition toward N toxicity (Shift E; Figure 5a). In contrast, P exhibited luxury consumption (Shift D) between P50 and P100, characterized by increases in both P concentration and content without a parallel rise in biomass. At higher P application rates, toxicity was evident through a decline in overall plant biomass (Figure 5b). The K deficiency observed under the control was mitigated by the P25 treatment (Figure 5c). From P25 to P50, K concentration remained stable while both biomass and K content increased, reflecting a state of K sufficiency (Shift B). Further increases in P shifted K status toward excess, characterized by toxic accumulation (Shift E) and antagonism (Shift F; Figure 5c).

## 3. Discussion

Seedling growth of *Homalium ceylanicum* increased with increasing P supply, reaching a maximum at 50 mg P per seedling. Above this level, tissue P concentration continued to increase without a corresponding improvement in growth. Such luxury P consumption has been reported previously [28]. At the highest P level, excessive P accumulation may have disrupted nutrient homeostasis and impaired nutrient acquisition and utilization, thereby suppressing seedling growth [29]. Zheng et al. reported that the overall growth of *Acacia melanoxylon* seedlings was greatest at 100 mg P per seedling from the fourth week of fertilization onward, whereas higher P supply provided no additional growth advantage [30]. Parks et al. found that the shoot dry weight of *Banksia ericifolia* increased with P supply up to 100 mg P L^−1^ substrate but declined at 200 mg P L^−1^ substrate due to P toxicity [31]. In comparison, the growth of *H. ceylanicum* declined when P supply exceeded 50 mg per seedling, suggesting a relatively narrow optimal P range and greater sensitivity to excessive P supply under exponential fertilization.

As a central constituent of energy carriers (e.g., ATP and NADPH), metabolic intermediates (e.g., sugar phosphates), and membrane phospholipids, P is indispensable to photosynthetic function and carbon metabolism [32]. In our study, P_n_ of *H. ceylanicum* seedlings displayed a unimodal response to P supply, increasing up to an optimum before declining at higher doses. This indicates that both deficient and excessive P availability can inhibit carbon assimilation, a phenomenon also reported in Arachis hypogaea [33]. Previous studies have linked such photosynthetic inhibition under non-optimal P supply to reductions in G_s_ and T_r_, as well as diminished sink strength for photoassimilates [34]. However, the contrasting trends observed among P_n_, G_s_, and C_i_ in our experiment suggest the involvement of non-stomatal limitations [35]. Variations in photosynthetic pigment contents (Chl a, Chl b and Car) closely mirrored the Pn trend, supporting the view that both P deficiency and excess impair the photosystem and elevate reactive oxygen species (ROS) levels [36]. Under P stress, the absorbed light energy often exceeds metabolic demand, leading to ROS accumulation and photodamage to photosystem II [37]. The declines in F_v_/F_m_, Y(II), ETR, and q_P_ under non-optimal P conditions confirmed the onset of photoinhibition in *H. ceylanicum*. The concomitant reduction in both F_v_/F_m_ and ETR further indicates compromised redox homeostasis and an insufficient capacity to mitigate oxidative stress, which corroborates earlier findings by Podgorska et al. [38] and Pashkovskiy et al. [39]. These results collectively underscore the importance of precise P management in sustaining photosynthetic efficiency and pigment stability during seedling development.

The concurrent increases in tissue P and N concentrations of *H. ceylanicum* were consistent with findings in *Senegalia senegal*, where exponential P addition promoted both P accumulation and mineral N acquisition [40]. Phosphorus is essential for ATP production and plays a key role in the metabolic pathways that drive active N uptake and assimilation [41]. In the modified Hoagland solution, N was supplied as both NO_3_^−^ and NH_4_^+^. Prior to amino acid biosynthesis, absorbed NO_3_^−^ is sequentially reduced to NO_2_^−^ and then NH_4_^+^, a process that requires metabolic energy and reducing power [42,43]. Thus, inadequate P supply may restrict N acquisition and assimilation by limiting energy-dependent steps [44], whereas excessive P may disturb nutrient homeostasis and suppress further N uptake [45]. These effects may explain why the greatest whole-plant N accumulation was observed under moderate P supply.

Vector analysis provided an integrated assessment of seedling nutritional status and its dynamic response to P fertilization. Within the 0–50 mg P range, the concurrent increases in biomass, nutrient concentrations, and whole-plant nutrient contents corresponded to Shifts B and C, indicating that increasing P supply alleviated P limitation and promoted effective nutrient loading in *H. ceylanicum* seedlings. Beyond 50 mg P, however, luxury P consumption (Shift D) became evident, accompanied by antagonistic effects on N and K uptake, indicative of incipient toxicity (Shift E). Such P-induced nutrient antagonism has been widely documented [46,47], suggesting that the 50–100 mg P range represents a transitional “nutrient loading–toxicity overlap zone” in *H. ceylanicum*. A notable trade-off was observed: the 50 mg P treatment supported maximum biomass with an optimal balance between growth and P accumulation, whereas 100 mg P promoted higher P reserves but triggered antagonistic effects on N and K. This overlap between enhanced P storage and incipient toxicity may be attributable to the relatively broad P intervals tested in this experiment. Future studies employing finer P gradients within the 50–100 mg range are needed to determine the optimal dosage that maximizes P reserves without causing significant nutrient antagonism.

The effectiveness of P fertilization is highly soil-dependent because soil pH, organic matter, texture, and P-fixation capacity govern phosphate solubility, sorption, diffusion, and root accessibility [48,49]. Such soil-dependent responses have also been observed in tropical tree seedlings. Wan Juliana et al. found that *Lagerstroemia floribunda* seedlings grew faster in soils with higher initial available P, whereas the response of specific leaf area to P addition was strongest in the P-poor laterised-shale soil [50]. Similarly, Nilus et al. showed that P addition promoted the growth of *Neolamarckia cadamba* seedlings in P-poor sandstone-hill soil but elicited little growth response in the more P-rich alluvial soil [51]. These findings demonstrate that initial soil P availability determines the extent to which P addition promotes seedling growth. In the present study, *H. ceylanicum* seedlings were cultivated in an acidic red loam–peat substrate with very low available P. Therefore, the optimum rate of 50 mg P per seedling identified here should be regarded as a substrate-specific reference for acidic, low-P nursery media rather than a universal recommendation. Before applying this protocol to other nurseries or field sites, soil or substrate testing is recommended, particularly for pH, available P, organic matter, texture, and P-fixation capacity. Although the optimal P dose identified here is specific to *H. ceylanicum* and the red loam–peat substrate used, the biphasic response to P supply—growth promotion under adequate P and nutrient antagonism under excessive P—may also be relevant to other tropical broad-leaved tree species grown under P-limited conditions. More broadly, integrating growth responses, photosynthetic performance, whole-plant nutrient accumulation, and vector analysis provides a transferable framework for identifying species-specific P-loading thresholds and avoiding excessive fertilization in tropical tree nurseries.

## 4. Materials and Methods

### 4.1. Plant Materials

This study was conducted in a greenhouse at the Research Institute of Tropical Forestry, Chinese Academy of Forestry, in Guangzhou, Guangdong Province, China (23°11′28″ N, 113°22′44″ E). In October 2023, seeds of *Homalium ceylanicum* were collected from plus trees in Meijiang District, Meizhou City, Guangdong Province (24°16′ N, 116°06′ E). After collection, the seeds were cleaned and surface-sterilized in 0.5% potassium permanganate (KMnO_4_) solution for 30 min and then soaked in distilled water for 6 h to promote germination. They were then sown in a sand bed maintained at 50–60% moisture content. After germination, seedlings at the 3–4 leaf stage were transplanted into plastic nursery pots (11.5 cm in top diameter × 11.5 cm in height) filled with a 4:1 (*v*/*v*) mixture of local red loam and peat and cultivated following standard nursery protocols. In July 2024, uniform, healthy seedlings (mean height: 16.73 ± 0.21 cm; mean root collar diameter: 2.26 ± 0.02 mm) were selected for the experiment. The initial chemical properties of the growing substrate are summarized in Table 3.

### 4.2. Experimental Design and Fertilization

A randomized complete block design with three replicates was employed in the experiment. Six P (Phosphorus) fertilization treatments were applied: 0 (control), 25, 50, 100, 200 and 300 mg P per seedling, designated as CK, P25, P50, P100, P200 and P300, respectively. Each experimental plot contained 25 seedlings, with 450 seedlings in total (6 treatments × 3 replicates × 25 seedlings). The initial whole-seedling nutrient contents were 14.73 ± 0.54 mg for N, 1.01 ± 0.04 mg for P and 13.30 ± 0.49 mg for K per seedling.

For the P25-P300 treatments, the total amount of P was supplied according to an exponentially increasing schedule, as prescribed by Equation (1) [52]:

(1) $$P_{T}=P_{S}[exp\left(rT\right)-1]$$

where *P*_T_ represents the target total amount of P to be supplied per seedling (ranging from 25 to 300 mg), *P*_S_ denotes the initial P content in the whole plant (1.01 ± 0.04 mg P per seedling) determined at the beginning of the experiment; *T* is the total number of fertilizer applications (*T* = 12); and *r* is the relative addition rate required to raise the initial P content (*P*_S_) to the predetermined target P content (*P*_T_). The value of *r* was first derived from Equation (1). Subsequently, the amount of P to be added at each application (*P*_t_) was calculated iteratively using Equation (2):

(2) $$P_{t}=P_{S}\;\left[\exp\left(rt\right)-1\right]-P_{t-1}$$

where *P*_t−1_ refers to the cumulative amount of P supplied before the current application, and *t* indicates the current application number. The detailed fertilization schedule is presented in Table 4.

A modified Hoagland nutrient solution (Coolaber Science & Technology Co., Ltd., Beijing, China; Cat. No. NS10205-P) formulated without P was used as the basal nutrient solution. P was supplied as sodium dihydrogen phosphate dihydrate (NaH_2_PO_4_·2H_2_O). At each fertilizer application, the amount of NaH_2_PO_4_·2H_2_O corresponding to the P dose in Table 4 was dissolved in 35 mL of the basal nutrient solution and applied to each seedling. Fertilization was conducted weekly for 12 weeks, from 23 July to 15 October 2024.

To prevent drought stress, nursery pots were weighed daily and watered as needed to maintain the substrate moisture at 50–70% of its maximum water-holding capacity, following the method described by Chen et al. [19]. Additionally, pot positions were randomized every two weeks throughout the experimental period to minimize potential edge effects.

### 4.3. Data Collection and Measurement

#### 4.3.1. Growth, Biomass, and Nutrient Measurements

Seedling height and root collar diameter (RCD) were measured for all seedlings before fertilization (week 0) and at 4, 8, and 12 weeks after the initiation of the fertilization treatments, corresponding to 20 August, 17 September, and 15 October, respectively. On 5 November 2024 (15 weeks after fertilization), six seedlings per plot were randomly selected, harvested, and separated into leaves, stems and roots. The components were oven-dried to constant weight using a two-stage process: they were first heated at 105 °C for 30 min and then dried at 60 °C for 72 h, after which the dry weight of each component was determined.

Based on these measurements, several growth parameters were derived. Total seedling biomass was calculated as the sum of the dry weights of leaves, stems, and roots; the root-to-shoot ratio (R/S) was defined as the ratio of root dry weight to the combined dry weight of stems and leaves. Additionally, the seedling quality index (*QI*) was calculated according to Dickson et al. [53] using Equation (3):

(3) QI=T/[(H/RCD)+(S/R)]

where *T* is the total seedling biomass (g), *H* is the seedling height (cm), *RCD* is the root collar diameter (mm), *S* is the shoot biomass (g), and *R* is the root biomass (g).

Following biomass determination, the dried leaf, stem, and root samples from the same seedlings were each ground separately into a homogeneous powder for nutrient analysis. Total N concentration was determined using the Kjeldahl method [54], total P concentration was analyzed via the vanadomolybdate yellow colorimetric method [55], and total K concentration was quantified by flame atomic absorption spectrophotometry [56]. The nutrient content of each organ (in milligrams) was calculated as the product of its nutrient concentration and corresponding dry weight, and the total seedling nutrient content was calculated as the sum of the nutrient contents of the roots, stems, and leaves.

#### 4.3.2. Photosynthetic Parameters and Leaf Area Measurements

Photosynthetic parameters and leaf area were quantified during the 15th week of the experiment, from 5 to 12 November 2024. Six seedlings per plot were randomly selected for evaluation. Gas exchange measurements were performed on sunny days between 9:00 and 11:00 a.m. using an LI-6800 portable photosynthesis system (Li-COR, Lincoln, NE, USA). The net photosynthetic rate (P_n_), intercellular CO_2_ concentration (C_i_), stomatal conductance (G_s_) and transpiration rate (T_r_) were measured at an ambient CO_2_ concentration of 400 μmol mol^−1^, a relative humidity of 70%, and photosynthetically active radiation (PAR) of 1100 μmol m^−2^ s^−1^ (the ratio of red to blue light was 9:1).

Subsequently, chlorophyll fluorescence was measured on the same seedlings with a PAM-2500 fluorometer (Heinz Walz, Effeltrich, Germany) after the leaves had been dark-adapted for one hour. The parameters assessed included the maximum photochemical quantum yield of photosystem II (F_v_/F_m_), the effective photochemical quantum yield of photosystem II [Y(II)], the coefficient of photochemical fluorescence quenching (q_P_), Stern–Volmer-type non-photochemical fluorescence quenching (NPQ), and the relative electron transport rate (ETR). All measurements were conducted on healthy, fully expanded, intact leaves situated near the apical region of the seedlings.

Following the gas exchange and chlorophyll fluorescence measurements, leaf area was determined on the same set of seedlings using a CI-203 handheld laser leaf area meter (CID Bio-Science, Camas, WA, USA). For each seedling, 15 mature, healthy, and uniformly sized leaves were sampled from the middle and upper parts of the seedling; if fewer than 15 suitable leaves were available, all representative leaves were collected and measured. Mean leaf area was calculated as the total leaf area divided by the number of leaves measured.

For photosynthetic pigment analysis, an additional six seedlings per plot were randomly selected. Leaf samples were collected between 9:00 and 10:00 a.m., and photosynthetic pigments were promptly extracted from the samples using 95% (*v*/*v*) ethanol. The absorbance of the extracts was measured at wavelengths of 649 nm, 665 nm, and 470 nm to determine the contents of chlorophyll a (Chl a), chlorophyll b (Chl b), and carotenoids (Car), respectively.

### 4.4. Statistical Analysis

All statistical analyses were performed with IBM SPSS 13.0 software. Differences in growth parameters, photosynthetic characteristics, and nutrient status across the various P treatments were evaluated by one-way analysis of variance (ANOVA). Tukey’s test was applied for post hoc multiple comparisons at the 0.05 significance level. The nutritional status of seedlings under different P treatments was assessed using vector analysis, following the methodology adapted from Salifu et al. [18] and as implemented in recent studies by Hong et al. and Feng et al. [12,57].

## 5. Conclusions

This study evaluated the effects of exponential P fertilization on the growth performance, photosynthetic characteristics, and nutritional status of one-year-old *Homalium ceylanicum* seedlings. The results revealed that adequate P supply enhanced photosynthetic carbon assimilation and biomass accumulation, whereas excessive P input disrupted nutrient homeostasis and suppressed whole-plant biomass production. Among the tested treatments, 50 mg P per seedling achieved the most favorable balance between growth promotion and nutrient loading, with whole-plant N accumulation increasing by 171.04% relative to the unfertilized control. Vector analysis further identified this dose as the optimal nutrient-loading threshold without excessive P accumulation or marked antagonistic effects on N and K. The findings provide a basis for nursery fertilization of this species and may also serve as a useful reference for similar tropical broad-leaved tree species. Further field validation and finer P gradient trials within the range of 50–100 mg P per seedling are warranted to refine this fertilization strategy.

## Figures and Tables

**Table 1:** Effects of different P application rates on chlorophyll fluorescence parameters of *Homalium ceylanicum* seedlings. Values are presented as means ± SE. Different lowercase letters within a column indicate significant differences among treatments according to Tukey’s test at the 0.05 level.

Treatment	F_v_/F_m_	Y(II)	ETR	q_P_	NPQ
CK	0.47 ± 0.01 c	0.03 ± 0.00 c	2.50 ± 0.06 c	0.12 ± 0.01 c	1.68 ± 0.03 a
P25	0.72 ± 0.04 ab	0.13 ± 0.00 b	11.40 ± 0.40 b	0.26 ± 0.01 b	1.55 ± 0.04 bc
P50	0.76 ± 0.02 a	0.17 ± 0.01 a	14.46 ± 0.31 a	0.34 ± 0.01 a	1.72 ± 0.01 a
P100	0.72 ± 0.04 ab	0.14 ± 0.00 b	11.80 ± 0.45 b	0.28 ± 0.02 ab	1.64 ± 0.01 ab
P200	0.71 ± 0.04 ab	0.14 ± 0.01 b	12.03 ± 0.52 b	0.27 ± 0.02 ab	1.50 ± 0.06 c
P300	0.64 ± 0.02 b	0.12 ± 0.00 b	10.68 ± 0.23 b	0.27 ± 0.02 ab	1.62 ± 0.03 abc

**Table 2:** Effects of different P application rates on N, P, and K concentrations in the roots, stems, and leaves of *Homalium ceylanicum* seedlings. Values are presented as means ± SE. Different lowercase letters within a column indicate significant differences among treatments according to Tukey’s test at the 0.05 level.

Treatment	Nutrient Concentration (mg g^−1^)								
	Root			Stem			Leaf		
	N	P	K	N	P	K	N	P	K
CK	6.50 ± 0.50 c	0.33 ± 0.02 d	7.32 ± 0.13 c	5.94 ± 0.62 c	0.24 ± 0.04 d	8.17 ± 1.00 b	16.63 ± 0.25 c	0.57 ± 0.01 d	11.77 ± 0.23 b
P25	7.71 ± 0.49 bc	0.64 ± 0.05 c	8.35 ± 0.25 bc	6.10 ± 0.36 c	0.63 ± 0.07 c	9.52 ± 0.43 ab	17.26 ± 0.03 c	1.07 ± 0.01 c	15.46 ± 0.27 a
P50	8.07 ± 0.38 ab	0.78 ± 0.06 bc	8.03 ± 0.42 bc	7.16 ± 0.56 b	0.82 ± 0.03 bc	10.61 ± 0.56 a	18.56 ± 0.36 b	1.33 ± 0.02 b	15.10 ± 0.47 a
P100	8.32 ± 0.62 ab	0.89 ± 0.06 ab	9.74 ± 0.14 ab	7.59 ± 0.36 ab	1.04 ± 0.14 ab	10.41 ± 0.96 ab	18.90 ± 0.18 b	1.48 ± 0.03 a	15.72 ± 0.14 a
P200	9.35 ± 0.42 a	0.83 ± 0.05 ab	10.46 ± 0.87 a	8.01 ± 0.36 ab	1.12 ± 0.07 a	11.88 ± 0.86 a	19.12 ± 0.20 b	1.55 ± 0.02 a	16.21 ± 0.37 a
P300	9.00 ± 0.30 ab	0.98 ± 0.05 a	8.76 ± 0.85 abc	8.67 ± 0.53 a	1.27 ± 0.09 a	10.98 ± 0.56 a	20.12 ± 0.50 a	1.57 ± 0.06 a	15.95 ± 0.24 a

**Table 3:** Initial chemical properties of the growing substrate used for *Homalium ceylanicum* seedling cultivation. Values are presented as mean ± SE.

pH	Organic Matter (g kg^−1^)	Total N (g kg^−1^)	Total P (g kg^−1^)	Total K (g kg^−1^)	Available N (mg kg^−1^)	Available P (mg kg^−1^)	Available K (mg kg^−1^)
4.91 ± 0.01	44.52 ± 0.10	0.97 ± 0.00	0.32 ± 0.00	9.15 ± 0.01	119.45 ± 0.43	2.33 ± 0.03	38.10 ± 0.11

**Table 4:** Weekly schedule of P application on *Homalium ceylanicum* seedlings.

Treatment	Amount of P Application (mg per Seedling)												
	1 wk	2 wk	3 wk	4 wk	5 wk	6 wk	7 wk	8 wk	9 wk	10 wk	11 wk	12 wk	Total
CK	0	0	0	0	0	0	0	0	0	0	0	0	0
P25	0.31	0.41	0.54	0.70	0.92	1.21	1.59	2.09	2.74	3.60	4.72	6.73	25
P50	0.39	0.54	0.74	1.03	1.45	1.97	2.77	3.84	5.33	7.40	10.27	14.27	50
P100	0.47	0.69	1.01	1.48	2.18	3.20	4.71	6.92	10.17	14.94	21.96	32.28	100
P200	0.55	0.86	1.34	2.09	3.25	5.05	7.87	12.24	19.06	29.66	46.17	71.86	200
P300	0.60	0.98	1.57	2.53	4.07	6.54	10.56	16.98	27.33	44.05	70.83	114.02	300

## Data Availability

The original contributions presented in this study are included in the article. Further inquiries can be directed to the corresponding author.
